# Prepregnancy Obesity and Risks of Stillbirth

**DOI:** 10.1371/journal.pone.0138549

**Published:** 2015-10-14

**Authors:** Suzan L. Carmichael, Yair J. Blumenfeld, Jonathan Mayo, Emily Wei, Jeffrey B. Gould, David K. Stevenson, Gary M. Shaw, March of Dimes Prematurity Research Center at Stanford University School of Medicine

**Affiliations:** 1 Department of Pediatrics, Stanford University School of Medicine, Stanford, California, United States of America; 2 Department of Obstetrics and Gynecology, Stanford University School of Medicine, Stanford, California, United States of America; University of Arkansas for Medical Sciences, UNITED STATES

## Abstract

**Background:**

We examined the association of maternal obesity with risk of stillbirth, focusing on whether the pattern of results varied by gestational age or maternal race-ethnicity or parity.

**Methods:**

Analyses included 4,012 stillbirths and 1,121,234 liveborn infants delivered in California from 2007–2010. We excluded stillbirths due to congenital anomalies, women with hypertensive disorders or diabetes, and plural births, to focus on fetuses and women without these known contributing conditions. We used Poisson regression to estimate relative risks (RR) and 95% confidence intervals (CI). Separate models were run for stillbirths delivered at 20–23, 24–27, 28–31, 32–36, 37–41 weeks, relative to liveborn deliveries at 37–41 weeks.

**Results:**

For stillbirth at 20–23 weeks, RRs were elevated for all race-ethnicity and parity groups. The RR for a 20-unit change in BMI (which reflects the approximate BMI difference between a normal weight and an Obese III woman) was 3.5 (95% CI 2.2, 5.6) for nulliparous white women and ranged from 1.8 to 5.0 for other sub-groups. At 24–27 weeks, the association was significant (p<0.05) only for multiparous non-Hispanic whites; at 28–31 weeks, for multiparous whites and nulliparous whites and blacks; at 32–36 weeks, for multiparous whites and nulliparous blacks; and at 37–41 weeks, for all groups except nulliparous blacks. The pattern of results was similar when restricted to stillbirths due to unknown causes and somewhat stronger when restricted to stillbirths attributable to obstetric causes.

**Conclusion:**

Increased risks were observed across all gestational ages, and some evidence of heterogeneity of the associations was observed by race-ethnicity and parity.

## Introduction

Stillbirth, *in utero* death at ≥20 weeks gestation, affects 5–6 per 1,000 deliveries in the US, and its prevalence is several times higher in lower-income countries [1]. It is devastating to families and substantially impacts parental mental health, resulting in high rates of depression and anxiety [2]. In recent decades, the overall prevalence of stillbirth has declined, but these gains are largely confined to later stillbirths (i.e., ≥28 weeks) and attributed to improvements in obstetric care and prevention of intrapartum complications [3, 4]. The prevalence of earlier stillbirths (i.e., <28 weeks), which comprise about half of all stillbirths, has not declined. Some risk factors for stillbirth have been identified, including obesity, nulliparity and black race-ethnicity, but in general our knowledge about its causes and how to prevent it is limited [5].

Obesity has been identified as one of the most important risk factors for stillbirth, largely due to how common it is and its potential modifiability [1]. We conducted a study of stillbirth and obesity within a study population of 1.2 million liveborn and stillborn infants delivered in California from 2007–2010. Our hypothesis was that the association between maternal obesity and risk of stillbirth varies by gestational age and maternal race-ethnicity and parity. Our analysis adds to existing literature in the following ways. First, we examine risk separately for different gestational age groupings, and we include stillbirths that encompass the earliest stillbirths reported in the U.S. (i.e., ≥20 weeks gestation). Most previous studies examine risks overall or focus on a truncated set of gestational ages. Second, we consider differences in results by race-ethnicity and parity, given known variability in stillbirth prevalence by race-ethnicity and a recent report indicating that the association of maternal obesity with liveborn preterm delivery (<37 weeks) varied by parity [6]. Third, we focus on women *without* diabetes or hypertensive disorders because we are primarily interested in the stillbirth-obesity association among women not already deemed high risk based on these additional conditions.

## Methods

This study includes data from deliveries in California from 2007–2010 obtained from vital records (i.e., fetal death and live birth certificates) and maternal and infant hospital discharge records, which were linked by the California Office of Statewide Health and Planning (OSHPD), as described previously [7]. Stillbirth in these records is defined as delivery at ≥20 weeks gestation. These birth years span the year that collection of maternal weight and height in vital records began (2007) and the most recent data year available at the time the analyses were conducted (2010).

Data derived from vital records include maternal pre-pregnancy weight and height, race-ethnicity, parity, education, and age, infant gestational age at delivery (based on the best obstetric estimate), and stillbirth cause of death. Data from hospital discharge records include maternal conditions based on reported ICD-9-CM diagnoses. Body mass index (BMI) was computed as pre-pregnancy weight divided by height-squared (kg/m^2^).

After excluding non-singleton deliveries and deliveries at <20 or >41 weeks or unknown gestation, there were 9,748 stillbirths and 2,062,230 live births, resulting in a prevalence of 4.7 stillbirths per 1,000 live births. Among these births, 80% of stillbirths (n = 7,800) and 97% of live births (n = 1,998,107) were linked with hospital discharge records. Stillbirths that were not linked to maternal discharge records were more likely to be delivered at very early gestational ages (20–23 weeks) than those that were linked. However, the distribution of maternal socio-demographic variables such as race-ethnicity, age and education were similar among linked and non-linked deliveries (data not shown).

Our comparison group was live births delivered at term (i.e., 37–41 weeks; n = 1,852,941). Preterm delivery is also an adverse outcome related to obesity; as such, inclusion of preterm deliveries in the comparison group could in theory result in under-estimation of the stillbirth-obesity association, especially at the earliest gestational ages, when it can be considered a competing outcome with stillbirth.[8]

We excluded subjects with congenital anomalies or chromosomal abnormalities (1,140 stillbirths, 128,493 live births) because they may have distinct etiologies. We excluded women with incalculable BMI due to missing height or weight reporting (587 stillbirths, 151,683 live births), outlier height <1.35 or >1.96 meters (13 stillbirths, 1,835 live births), outlier weight <34.02 or >204.12 kg (25 live births), or who were underweight (BMI<18.5 kg/m^2^: 209 stillbirths, 64,734 live births); and women who were not white, black or Hispanic, due to small sample sizes (713 stillbirths, 211,176 live births). Among these deliveries that were eligible for analysis, 4,920 (out of 5,138) stillbirths and 1,271,080 (out of 1,294,995) live births had complete data on covariates.

We also excluded women with pre-gestational diabetes (ICD-9 codes 250 and 648.0), chronic hypertension (401–405, 642.0, 642.1, 642.2, 642.7, and 642.9), gestational hypertension (642.3) or preeclampsia/eclampsia (642.4, 642.5, and 642.6) (728 stillbirths, 80,409 live births) [9]. We excluded women with gestational diabetes only from models examining risk of stillbirth at ≥24 weeks (ICD-9 code 648.8; 180 stillbirths, 69,437 live births), given that 20–23 weeks precedes the time that this condition is typically diagnosed (24–28 weeks). These exclusions were motivated by our goal to determine whether obesity alone, i.e., in the absence of these conditions, was associated with stillbirth. The exclusions resulted in mothers of 4,012 cases and 1,121,234 controls being included in our main analyses.

We grouped stillbirths according to cause of death listed on the fetal death certificate, following categories established by the NICHD-sponsored Stillbirth Collaborative Research Network as closely as possible [10]. The groups were cord complications (e.g., P02.5, compression of umbilical cord; P02.6, other conditions of cord); obstetric etiologies (e.g., P07.2, extreme immaturity; and P01.1, premature rupture of membranes); placental disorders (e.g., P02.1, placental separation and hemorrhage); fetal conditions (e.g., P29.1 cardiac dysrhythmia; P83.2 hydrops fetalis not due to haemolytic disease); infection (e.g., P02.7 chorioamnionitis); maternal conditions (e.g., P00.8, other maternal conditions); and other (e.g., P04.4, maternal use of drugs of addiction; P00.5, maternal injury). We examined the distribution of these groups by gestational age and BMI category (normal, overweight, obese).

Associations between BMI and stillbirth were estimated with relative risks (RRs) and their 95% confidence intervals via Poisson regression. Separate models were run for five gestational age groups of stillbirths (20–23, 24–27, 28–31, 32–36, 37–41 weeks), relative to liveborn term deliveries (37–41 weeks). We examined risks based on BMI as a continuous variable and as a quadratic (BMI-squared) to assess linearity or non-linearity of the associations, respectively. Given that BMI categories have practical clinical significance, we also examined BMI categorized as normal (18.5–24.9 kg/m^2^), overweight (25.0–29.9 kg/m^2^), obese class I (BMI 30.0–34.9 kg/m^2^), obese class II (BMI 35.0–39.9 kg/m^2^), and obese class III (BMI ≥40.0 kg/m^2^), based on NIH/NHLBI guidelines (http://www.nhlbi.nih.gov/health/public/heart/obesity/lose_wt/bmi_dis.htm). For continuous models, we calculated RRs for increments of 5-unit changes in BMI to roughly represent changes from one BMI category to the next, relative to normal BMI.

We decided *a priori* to stratify and present all analyses by parity (nulliparous versus multiparous) and race-ethnicity (non-Hispanic white, non-Hispanic Black, Hispanic), for reasons stated in the Introduction, but we did also conduct a formal test for interaction within each gestational age group, using product terms, for models that specified BMI as a continuous variable. We included the following potential confounders as reported in vital records in all models, based on *a priori* knowledge: maternal education (less than 12 years; high school graduate; some college; and college graduate or more), age (years), and height (to reduce potential residual confounding associated with the BMI algorithm) [11].

We conducted sensitivity analyses that included (rather than excluded) women with diabetes or hypertensive disorders and analyses of 20–23 week stillbirths that included those that were not linked with maternal discharge records (since most unlinked stillbirths are in this group).

We also examined categories of cause of death by gestational age and BMI group and conducted separate Poisson regression analyses restricted to stillbirths with the two most common categories of cause of death–obstetric and NOS (not otherwise specified). All analyses were conducted using SAS version 9.3 (Cary, NC). Approval was obtained from the California Committee for the Protection of Human Subjects and Stanford University IRB; analyses were based on de-identified, publicly available data and therefore informed consent was not obtained.

## Results

Of the stillbirth cases, 43% percent were delivered at <28 weeks gestation (Table 1). Mothers of stillborn infants were more likely to be obese, non-Hispanic black, nulliparous, and have lower education than mothers of term liveborn infants (p<0.001 for all comparisons; Table 1).

**Table 1:** Characteristics of stillbirths and liveborn term births included in analyses, California 2007–2010.^1^

	Stillbirths (n = 4,012)	Liveborn term births (n = 1,121,234) ^2^
Prepregnancy BMI (kg/m ^2^ )		
Normal (18.5–24.9)	1,702 (42.4)	584,370 (52.1)
Overweight (25.0–29.9)	1,205 (30.0)	312,941 (27.9)
Obese I (30.0–34.9)	643 (16.0)	143,387 (12.8)
Obese II (35.0–39.9)	283 (7.1)	53,211 (4.7)
Obese III (≥40.0)	179 (4.5)	27,325 (2.4)
Race-ethnicity		
Non-Hispanic White	1,162 (29.0)	368,925 (32.9)
Non-Hispanic Black	496 (12.4)	66,847 (6.0)
Hispanic	2,354 (58.7)	685,462 (61.1)
Parity		
Nulliparous	1,681 (41.9)	425,877 (38.0)
Multiparous	2,331 (58.1)	695,357 (62.0)
Age (years)		
<20	491 (12.2)	116,726 (10.4)
20–24	902 (22.5)	274,414 (24.5)
25–29	1,060 (26.4)	312,730 (27.9)
30–34	866 (21.6)	253,984 (22.7)
≥35	693 (17.3)	163,380 (14.6)
Education		
Less than high school graduate	1,360 (33.9)	319,151 (28.5)
High school graduate	1,121 (27.9)	315,919 (28.2)
Some college	981 (24.5)	259,728 (23.2)
≥ 4-year college degree	550 (13.7)	226,436 (20.2)
Gestational age at delivery		
20–23 weeks	1,154 (28.8)	
24–27 weeks	570 (14.2)	
28–31 weeks	525 (13.1)	
32–36 weeks	835 (20.8)	
37–41 weeks	928 (23.1)	1,121,234 (100)

^1^ p-values were <0.001 for Chi-square tests comparing each maternal characteristic among stillbirths versus liveborn term births.

^2^ This column represents term births that were included in analyses that excluded women with diabetes and hypertensive disorders. Gestational diabetes was not an exclusion criterion for models that included stillbirths delivered at 20–23 weeks; the number of term births for those comparison was 1,190,671.

For stillbirth at 20–23 weeks, RRs reflecting the association of BMI as a continuous variable ranged from 1.33 to 2.22 for a 10-unit change in BMI; confidence intervals excluded 1.0 for all race-ethnicity and parity groups, with RRs tending to be somewhat stronger among nulliparous than parous women (Table 2). The interaction term was significant for parity (p<0.01) but not for race-ethnicity (p = 0.38).

**Table 2:** Association of stillbirth with maternal pre-pregnancy body mass index specified as a continuous variable, by gestational age, race-ethnicity and parity, California 2007–2010.^1^

	Change in BMI	20–23 weeks	24–27 weeks	28–31 weeks	32–36 weeks	37–41 weeks
		RR (CI)	RR (CI)	RR (CI)	RR (CI)	RR (CI)
**Nulliparous**						
Non-Hispanic White	1-unit	**1.06 (1.04,1.09)**	1.03 (0.99,1.07)	**1.07 (1.03,1.11)**	1.03 (0.99,1.06)	**1.11 (1.05,1.17)** ^2^
	5-unit	**1.36 (1.21,1.54)**	1.15 (0.93,1.40)	**1.39 (1.16,1.65)**	1.13 (0.95,1.35)	**1.57 (1.26,1.95)**
	10-unit	**1.86 (1.47,2.36)**	1.31 (0.87,1.97)	**1.92 (1.35,2.72)**	1.29 (0.91,1.82)	**2.11 (1.52,2.93)**
	15-unit	**2.54 (1.78,3.63)**	1.50 (0.82,2.77)	**2.66 (1.58,4.48)**	1.46 (0.86,2.46)	**2.45 (1.59,3.76)**
	20-unit	**3.47 (2.15,5.59)**	1.72 (0.76,3.89)	**3.68 (1.83,7.39)**	1.65 (0.82,3.32)	**2.44 (1.22,4.86)**
Non-Hispanic Black	1-unit	**1.04 (1.01,1.07)**	1.00 (0.96,1.06)	**1.05 (1.00,1.10)**	**1.04 (1.00,1.09)**	1.03 (0.99,1.08)
	5-unit	**1.22 (1.06,1.42)**	1.02 (0.80,1.32)	**1.29 (1.01,1.63)**	**1.24 (1.01,1.52)**	1.18 (0.93,1.49)
	10-unit	**1.50 (1.12,2.01)**	1.05 (0.63,1.74)	**1.65 (1.03,2.67)**	**1.53 (1.02,2.32)**	1.39 (0.87,2.21)
	15-unit	**1.84 (1.18,2.86)**	1.07 (0.50,2.29)	**2.13 (1.04,4.35)**	**1.90 (1.02,3.53)**	1.63 (0.81,3.29)
	20-unit	**2.25 (1.25,4.06)**	1.10 (0.40,3.02)	**2.73 (1.05,7.11)**	**2.35 (1.03,5.37)**	1.92 (0.75,4.90)
Hispanic	1-unit	**1.08 (1.07,1.10)**	1.01 (0.98,1.04)	1.00 (0.97,1.04)	1.01 (0.98,1.04)	**1.04 (1.02,1.07)**
	5-unit	**1.49 (1.38,1.61)**	1.05 (0.88,1.24)	1.01 (0.84,1.22)	1.07 (0.92,1.24)	**1.22 (1.08,1.39)**
	10-unit	**2.22 (1.90,2.59)**	1.10 (0.78,1.54)	1.03 (0.70,1.50)	1.15 (0.85,1.54)	**1.50 (1.17,1.92)**
	15-unit	**3.31 (2.63,4.18)**	1.15 (0.69,1.92)	1.04 (0.59,1.83)	1.23 (0.79,1.90)	**1.84 (1.26,2.67)**
	20-unit	**4.94 (3.63,6.72)**	1.20 (0.61,2.39)	1.05 (0.50,2.25)	1.31 (0.73,2.36)	**2.25 (1.36,3.71)**
**Multiparous**						
Non-Hispanic White	1-unit	**1.04 (1.01,1.06)**	**1.06 (1.03,1.09)**	**1.04 (1.01,1.08)**	**1.04 (1.01,1.06)**	**1.13 (1.06,1.20)** ^2^
	5-unit	**1.20 (1.06,1.36)**	**1.36 (1.19,1.56)**	**1.24 (1.05,1.46)**	**1.19 (1.04,1.36)**	**1.63 (1.29,2.06)**
	10-unit	**1.44 (1.12,1.84)**	**1.85 (1.41,2.43)**	**1.54 (1.11,2.14)**	**1.42 (1.09,1.86)**	**2.03 (1.45,2.84)**
	15-unit	**1.73 (1.19,2.50)**	**2.51 (1.67,3.79)**	**1.91 (1.17,3.13)**	**1.69 (1.13,2.53)**	**1.92 (1.22,3.01)**
	20-unit	**2.07 (1.26,3.40)**	**3.41 (1.98,5.90)**	**2.38 (1.23,4.58)**	**2.02 (1.18,3.44)**	**1.38 (0.64,2.96)**
Non-Hispanic Black	1-unit	**1.04 (1.01,1.07)**	1.01 (0.97,1.06)	1.00 (0.96,1.05)	1.02 (0.99,1.06)	**1.18 (1.03,1.34)** ^2^
	5-unit	**1.19 (1.03,1.38)**	1.07 (0.86,1.33)	1.01 (0.80,1.28)	1.12 (0.94,1.34)	**1.91 (1.15,3.17)**
	10-unit	**1.43 (1.06,1.91)**	1.14 (0.74,1.77)	1.02 (0.63,1.64)	1.26 (0.88,1.79)	**2.41 (1.17,4.96)**
	15-unit	**1.71 (1.10,2.65)**	1.22 (0.64,2.36)	1.03 (0.51,2.10)	1.41 (0.83,2.40)	**2.00 (0.85,4.70)**
	20-unit	**2.04 (1.13,3.66)**	1.31 (0.55,3.13)	1.04 (0.40,2.69)	1.59 (0.78,3.21)	**1.09 (0.29,4.10)**
Hispanic	1-unit	**1.03 (1.01,1.05)**	1.00 (0.97,1.02)	1.01 (0.99,1.04)	1.00 (0.98,1.02)	**1.03 (1.01,1.04)**
	5-unit	**1.15 (1.07,1.25)**	0.98 (0.85,1.12)	1.05 (0.93,1.20)	0.98 (0.89,1.09)	**1.13 (1.04,1.23)**
	10-unit	**1.33 (1.13,1.56)**	0.95 (0.73,1.25)	1.11 (0.87,1.43)	0.96 (0.79,1.18)	**1.28 (1.08,1.52)**
	15-unit	**1.54 (1.21,1.96)**	0.93 (0.62,1.39)	1.17 (0.81,1.71)	0.95 (0.70,1.28)	**1.45 (1.12,1.88)**
	20-unit	**1.77 (1.29,2.44)**	0.91 (0.53,1.55)	1.24 (0.75,2.04)	0.93 (0.62,1.39)	**1.65 (1.17,2.33)**

^1^ Relative Risks (RR) reflect estimated risk of stillbirth relative to term (37–41 weeks) live birth adjusted for maternal age, education, and height. Each 5-unit change in BMI reflects the approximate difference in risk between the following categories of BMI: a 5-unit change represents the approximate difference in risk between women with normal BMI (18.5–24.9 kg/m^2^, with 22.5 taken as the approximate mid-point) versus overweight (25.0–29.9 kg/m^2^, with 27.5 as the mid-point); a 10-unit change, the difference between women with normal BMI and obese class I (BMI 30.0–34.9 kg/m^2^, with 32.5 as the mid-point); a 15-unit change, the difference between normal BMI and obese class II (BMI 35.0–39.9 kg/m^2^, with 37.5 as mid-point); and a 20-unit change, the difference between normal BMI and obese class III (BMI ≥40.0 kg/m^2^, with 42.5 as reference). Analyses exclude women with gestational or pre-gestational diabetes or pregnancy-induced or chronic hypertension with one exception. For estimates among stillbirth at 20–23 weeks, the comparison group of term live births included the following additional births beyond those shown in Table 1 who had gestational diabetes: non-Hispanic White (n = 386,490), non-Hispanic Black (n = 69,633), and Hispanic (n = 734,548).

^2^ The quadratic term (BMI-squared) was significant (p<0.10) for the noted models; this is an indication that the association between BMI and risk of stillbirth was not linear, as reflected in the RRs for these groups, which are all relative to a BMI of 22.5 kg/m^2^.

At 24–27 weeks, confidence intervals excluded 1.0 only for multiparous non-Hispanic whites (RR 1.85 for a 10-unit change in BMI); at 28–31 weeks, only for nulliparous whites and blacks and multiparous whites (RRs 1.92, 1.65 and 1.54, respectively, for a 10-unit change in BMI); and at 32–36 weeks, for nulliparous blacks and multiparous whites (RRs 1.53 and 1.42, respectively). RRs for the other groups tended to be closer to one. For all three gestational age groups, the interaction term was significant for race-ethnicity (p<0.05) but not for parity (p>0.3).

At 37–41 weeks, the association was linear and confidence intervals excluded 1.0 for nulliparous and multiparous Hispanics (RRs 1.50 and 1.28, respectively, for a 10-unit change in BMI). There was evidence of a non-linear association (i.e., p<0.10 for BMI-squared) for nulliparous and multiparous non-Hispanic whites and multiparous blacks (RRs were 2.11, 2.03, and 2.41, respectively, for a 10-unit change in BMI). Non-linear associations tended to suggest a weakening of the association at the highest levels of BMI (i.e., RRs did not continue to increase with increasing BMI). The interaction term was significant for parity (p<0.10) but not for race-ethnicity (p = 0.33).

Results for BMI as a categorical variable largely parallel the pattern revealed by BMI as a continuous variable (Table 3). RRs tended to be largest for stillbirth at 20–23 weeks and at 37–41 weeks and larger for nulliparous than multiparous women, but many confidence intervals included one.

**Table 3:** Association of stillbirth with categories of maternal pre-pregnancy body mass index by gestational age, race-ethnicity and parity, California 2007–2010.^1^

	BMI	20–23 weeks		24–27 weeks		28–31 weeks		32–36 weeks		37–41 weeks	
		N	RR (CI)	N	RR (CI)	N	RR (CI)	N	RR (CI)	N	RR (CI)
**Nulliparous**											
Non-Hispanic White	Normal	78	ref	46	ref	36	ref	65	ref	78	ref
	Overweight	28	1.06 (0.68,1.63)	22	1.49 (0.89,2.47)	18	1.54 (0.87,2.72)	26	1.26 (0.80,1.99)	32	1.25 (0.83,1.89)
	Obese I	24	**2.28 (1.44,3.61)**	4	0.74 (0.26,2.05)	13	**2.98 (1.57,5.65)**	11	1.47 (0.77,2.80)	28	**2.92 (1.89,4.52)**
	Obese II	8	1.91 (0.92,3.96)	4	1.92 (0.69,5.37)	4	2.42 (0.85,6.83)	6	2.10 (0.90,4.86)	6	1.63 (0.71,3.76)
	Obese III	10	**4.46 (2.29,8.66)**	2	–	2	–	1	–	5	**2.70 (1.09,6.69)**
Non-Hispanic Black	Normal	34	ref	19	ref	16	ref	17	ref	18	ref
	Overweight	21	1.26 (0.73,2.17)	12	1.31 (0.63,2.71)	8	1.14 (0.49,2.67)	17	**2.11 (1.07,4.13)**	9	1.03 (0.46,2.30)
	Obese I	13	1.67 (0.88,3.17)	4	0.95 (0.32,2.79)	3	0.98 (0.28,3.37)	5	1.39 (0.51,3.78)	6	1.55 (0.61,3.92)
	Obese II	10	**2.73 (1.34,5.57)**	6	**3.12 (1.23,7.89)**	2	–	2	–	2	–
	Obese III	6	2.36 (0.98,5.66)	0	–	4	**4.97 (1.63,15.1)**	4	**3.88 (1.30,11.7)**	3	2.65 (0.77,9.09)
Hispanic	Normal	105	ref	67	ref	61	ref	84	ref	85	ref
	Overweight	103	**2.02 (1.54,2.66)**	35	1.12 (0.74,1.68)	29	1.01 (0.65,1.58)	56	**1.45 (1.03,2.03)**	50	1.23 (0.87,1.75)
	Obese I	63	**2.96 (2.16,4.05)**	12	0.95 (0.52,1.77)	8	0.69 (0.33,1.44)	16	1.02 (0.60,1.75)	23	1.39 (0.87,2.20)
	Obese II	23	**3.12 (1.98,4.92)**	5	1.20 (0.48,2.98)	5	1.28 (0.51,3.21)	4	0.78 (0.28,2.12)	9	1.60 (0.80,3.18)
	Obese III	22	**6.09 (3.83,9.69)**	4	2.05 (0.74,5.65)	2	–	3	1.25 (0.40,3.98)	9	**3.35 (1.68,6.69)**
**Multiparous**											
Non-Hispanic White	Normal	67	ref	45	ref	34	ref	61	ref	61	ref
	Overweight	38	1.27 (0.85,1.89)	29	1.54 (0.96,2.47)	21	1.43 (0.83,2.47)	29	1.03 (0.66,1.61)	54	**2.11 (1.46,3.05)**
	Obese I	20	1.44 (0.87,2.39)	10	1.22 (0.61,2.44)	16	**2.43 (1.33,4.45)**	14	1.07 (0.60,1.92)	20	**1.79 (1.07,2.98)**
	Obese II	12	**1.96 (1.05,3.65)**	10	**2.87 (1.43,5.75)**	6	2.14 (0.89,5.15)	16	**2.82 (1.61,4.92)**	15	**3.14 (1.77,5.57)**
	Obese III	7	1.93 (0.88,4.23)	9	**4.62 (2.23,9.58)**	4	2.51 (0.88,7.16)	5	1.52 (0.61,3.79)	2	–
Non-Hispanic Black	Normal	22	ref	13	ref	15	ref	20	ref	13	ref
	Overweight	19	1.13 (0.61,2.10)	15	1.64 (0.78,3.45)	11	1.03 (0.47,2.26)	16	1.12 (0.58,2.16)	10	1.10 (0.48,2.50)
	Obese I	15	1.56 (0.81,3.02)	5	1.00 (0.36,2.81)	7	1.21 (0.49,2.97)	15	1.91 (0.97,3.74)	14	**2.83 (1.33,6.05)**
	Obese II	10	2.09 (0.99,4.42)	8	**3.23 (1.33,7.82)**	4	1.41 (0.47,4.26)	3	0.78 (0.23,2.65)	2	–
	Obese III	7	1.89 (0.81,4.44)	1	–	2	–	5	1.77 (0.66,4.74)	3	1.71 (0.48,6.01)
Hispanic	Normal	124	ref	70	ref	78	ref	144	ref	126	ref
	Overweight	132	1.22 (0.95,1.56)	63	1.09 (0.77,1.53)	56	0.87 (0.62,1.23)	112	0.92 (0.72,1.18)	134	1.28 (1.00,1.63)
	Obese I	78	**1.34 (1.01,1.79)**	37	1.26 (0.85,1.88)	42	1.28 (0.88,1.87)	50	0.81 (0.59,1.12)	67	1.26 (0.93,1.69)
	Obese II	32	**1.49 (1.01,2.20)**	13	1.24 (0.68,2.24)	14	1.23 (0.70,2.18)	16	0.75 (0.45,1.26)	26	1.40 (0.92,2.13)
	Obese III	23	**2.09 (1.34,3.27)**	0	–	4	0.73 (0.27,2.00)	12	1.18 (0.65,2.12)	18	**2.00 (1.22,3.28)**

^1^ Relative Risks (RR) reflect estimated risk of stillbirth relative to term (37–41 weeks) live birth adjusted for maternal age, education, and height; excludes women with gestational or pre-gestational diabetes or pregnancy-induced or chronic hypertension with one exception. For estimates among stillbirth at 20–23 weeks, the comparison group of term live births included the following additional births beyond those shown in Table 1 who had gestational diabetes: non-Hispanic White (n = 386,490), non-Hispanic Black (n = 69,633), and Hispanic (n = 734,548). RRs were not calculated for cells with fewer than 3 cases.

Categories of cause of death varied by gestational age and BMI. For fetal deaths at 20–23 weeks, 12–22% were NOS depending on BMI category. The most common cause was obstetric, which increased with increasing BMI, ranging from 43% for normal weight women to 64% for obese III women. For fetal deaths at >23 weeks gestation, 35–40% were NOS, for most groups (Table 4). Cord complications tended to increase with increasing gestation, regardless of BMI group.

**Table 4:** Distribution of cause of death among stillbirths, by gestational age at delivery and maternal BMI (n = 4,012).

BMI Category	Cause of Fetal Death Category	20–23 weeks(n = 1,154)	24–27 weeks(n = 570)	28–31 weeks(n = 525)	32–36 weeks(n = 835)	37–41 weeks(n = 928)	Overall
Normal BMI (n = 1,702)	Not otherwise specified	22%	40%	40%	39%	39%	35%
	Obstetric	43%	14%	8%	4%	3%	16%
	Placental	12%	13%	15%	16%	15%	14%
	Cord complications	7%	18%	25%	25%	31%	21%
	Fetal conditions	7%	8%	8%	8%	6%	7%
	Infection	7%	3%	1%	4%	3%	4%
	Maternal conditions	<1%	2%	2%	3%	1%	2%
	Other	2%	1%	2%	2%	1%	1%
Overweight (n = 1,205)	Not otherwise specified	18%	39%	36%	36%	38%	32%
	Obstetric	53%	16%	8%	7%	4%	21%
	Placental	10%	10%	14%	16%	13%	12%
	Cord complications	5%	19%	30%	29%	37%	23%
	Fetal conditions	7%	9%	7%	5%	5%	6%
	Infection	6%	3%	1%	2%	2%	3%
	Maternal conditions	1%	1%	1%	4%	1%	2%
	Other	1%	3%	3%	1%	1%	1%
Obese I (n = 643)	Not otherwise specified	13%	38%	35%	48%	34%	30%
	Obstetric	57%	17%	11%	5%	4%	24%
	Placental	7%	14%	27%	16%	13%	14%
	Cord complications	8%	24%	18%	19%	37%	20%
	Fetal conditions	8%	7%	4%	6%	6%	6%
	Infection	6%	0%	2%	4%	3%	4%
	Maternal conditions	2%	0%	2%	1%	3%	2%
	Other	0%	1%	0%	1%	1%	<1%
Obese II (n = 283)	Not otherwise specified	12%	43%	40%	43%	42%	32%
	Obstetric	62%	9%	23%	6%	0%	26%
	Placental	5%	11%	6%	6%	18%	9%
	Cord complications	2%	20%	23%	26%	33%	18%
	Fetal conditions	8%	9%	3%	15%	5%	8%
	Infection	8%	4%	0%	2%	2%	4%
	Maternal conditions	1%	4%	3%	0%	0%	1%
	Other	1%	0%	3%	2%	0%	1%
Obese III (n = 179)	Not otherwise specified	19%	19%	50%	33%	50%	31%
	Obstetric	64%	44%	6%	13%	8%	35%
	Placental	4%	6%	17%	23%	5%	9%
	Cord complications	7%	19%	11%	23%	28%	16%
	Fetal conditions	3%	6%	6%	3%	0%	3%
	Infection	3%	6%	11%	3%	5%	4%
	Maternal conditions	1%	0%	0%	0%	3%	1%
	Other	0%	0%	0%	0%	3%	1%

We ran models restricted to stillbirths with NOS and obstetric causes of death since they were the most common. The general pattern of results was that obesity was associated with substantially increased risk for both groups, with RRs tending to be somewhat higher for stillbirths with obstetric causes of death, relative to the overall results (S1 Table). We also ran models that included women with diabetes or hypertensive disorders. The general pattern of results was similar to models that excluded them (S2 Table). We ran models for stillbirths at 20–23 weeks (n = 1,263 in primary analyses), adding in stillbirths that were not linked to maternal discharge records (n = 823); results were similar to those that excluded them (data not shown). This work was approved by the California Committee for the Protection of Human Subjects.

## Discussion

In this study of more than 1.2 million California births, increasing level of maternal obesity was associated with increasing risk of stillbirth. Increased risks were observed across all gestational ages. Some heterogeneity by race-ethnicity and parity was observed but varied by gestational age. The pattern of results was somewhat stronger when restricted to stillbirths attributable to obstetric causes (which were primarily early delivery and premature rupture of membranes). These results were observed among women who did not have hypertensive disorders or diabetes; however, addition of women with these conditions to the analyses resulted in a similar pattern of results.

A recent meta-analysis of cohort studies of obesity and stillbirth reported a summary RR 1.24 (95%CI, 1.18–1.30) per 5-unit change in BMI [12], which is within the range of the magnitude of risk that we observed for a 5-unit change, among sub-groups that had a significant association. Previous studies of obesity and stillbirth have tended to focus on the overall association regardless of factors such as gestational age or cause of death, with a few notable exceptions. A recent large study of vital records in Texas and Washington reported that increasing level of maternal obesity was associated with increasing risk of stillbirth, especially among term deliveries, after adjustment for factors such as maternal parity, race-ethnicity, and pre-pregnancy hypertension and diabetes as potential confounders [13]. Proportional hazards models only included stillbirths at 30 weeks or later, although they comprised 45% of all cases. Nohr et al. examined risk by gestational age and cause of death, but their ability to discern differences was limited by small sample size (about 150 stillbirths) and restriction to stillbirths at 28 weeks or later. We are unaware of other studies of stillbirth and obesity that evaluated differences by gestational age (particularly the early gestational ages) or cause of death. Another important feature of our analysis is its focus on results that exclude women with diabetes or hypertensive disorders, rather than treating these important co-morbidities as confounders or ignoring them. We are aware of two other studies that used our approach, and their results were similar to ours [14, 15], in that results were similar whether these women were included or excluded.

Our study is also unique in its presentation of results stratified by race-ethnicity and parity. A study by Salihu et al. reported that the association of obesity with risk of stillbirth was slightly stronger among black than white women [16]. We are unaware of other studies that examined differences by race-ethnicity or parity. We observed some evidence for differences in the obesity-stillbirth association by race-ethnicity and parity, but results varied across the gestational age spectrum. Variability by race-ethnicity or parity in the frequency of unmeasured underlying etiologic factors as well as clinical management could contribute to differences in associations by race-ethnicity and parity. More in-depth studies are needed to explain the observed differences.

The exclusions and stratifications that we implemented resulted in analysis of a sub-set of the population. Our approach could compromise generalizability to the entire study population, but this trade-off was acceptable to us in that it enabled us to investigate more specific etiologic questions regarding the obesity-stillbirth association.

For the analytic framework, we chose to parallel a nested case-control design with the optimal outcome being term live birth. We chose this approach because in theory, results from a proportional hazards model could be biased because preterm live birth represents censoring of an adverse outcome that is related to the exposure of interest and also likely shares common mechanisms with stillbirth, and furthermore the extent of this censoring likely varies over the course of gestation (e.g., in this population the obesity/preterm association is strongest at the earliest gestational ages). The situation is complicated by the fact that the *definition* of preterm delivery is based solely on time. Pragmatically, however, the extent of bias incurred by using one approach versus another is likely to be small because stillbirth and liveborn preterm delivery are so much less prevalent than liveborn term delivery.

We recently conducted a study of the association of maternal obesity with liveborn preterm delivery, using the same study population as used for the current study [6]. The strongest association was for the earliest preterm deliveries (20–23 weeks) among nulliparous women. Associations were considerably weaker or non-existent for later preterm delivery and among multiparous women, and this pattern of results was similar by race-ethnicity. Our current analysis parallels these findings, in that somewhat stronger risks were observed among nulliparous than parous women for the earliest stillbirths, but it differs in that substantially increased risks were also observed among parous women.

Obesity and stillbirth are both complex, and many potential factors may contribute to their association. Stillbirth may stem from a variety of adverse conditions, including placental insufficiency, preterm onset of labor or rupture of membranes, infection and cord abnormalities. Obesity could contribute to any of these problems. In addition, obesity may contribute to lower sensitivity with regard to detection of fetal complications, on the part of monitoring tools or maternal ability to detect changes in fetal movement.

Our study has several strengths. It included a large diverse study population and separate analysis by gestational age, cause of death, and maternal race-ethnicity, parity, hypertensive disorders and diabetes. It used hospital discharge data linked with vital records, which provides a more complete reporting of maternal conditions than either resource alone [17, 18]. We excluded infants for whom congenital anomalies were reported on vital records or as the underlying cause of stillbirth. This is important given that these cases may have distinct etiologies and may be more likely to reflect fetal rather than maternal or placental-based etiologies. Although using hospital discharge data in conjunction with vital records has strengths (e.g., it is highly cost-efficient), the information is still more limited than detailed medical records. For example, we had to rely on self-reported pre-pregnancy weight and height. Measurement error in these factors is not expected to vary based on pregnancy outcome, but it is expected to vary by BMI, in that higher-BMI women are more likely to under-report their weight [19]. This error would misclassify these women into lower BMI categories, which would likely contribute to under-estimation of risk. We excluded underweight women so we could focus on the impact of higher BMI on stillbirth risk, but this is an important group that merits further investigation. We excluded post-term births (i.e., >41 weeks) because such deliveries are rare and usually preventable with appropriate obstetric management; <1% of stillbirths in California are post-term. We excluded women with diabetes or hypertensive disorders for reasons stated above; prior studies indicate that sensitivity and specificity is high for maternal diabetes and hypertensive disorders derived from hospital discharge data [17, 18] although we acknowledge that it is inferior to actual medical chart review. We relied on fetal death certificates for information on cause of death; as such, these results an interesting and important [12] adjunct to our analyses but should be interpreted with caution. The accuracy and completeness of cause of death on fetal death certificate is known to be limited [18, 20]. As noted above, the cause of death was NOS for 35–40% of stillbirths. More thorough and validated information on cause of death would have been helpful, although even with the most intense level of investigation, cause of death would still likely remain unknown for a substantial proportion of stillbirths. For example, the cause of 24% of stillbirths remained unknown in the Stillbirth Collaborative Research Network (SCRN) study, which involved intense data collection and evaluation (autopsy, placental exam, clinical tests) [10]. The distribution of causes of death in our study has additional differences from the SCRN, but this is not surprising given our exclusions and the fact that the SCRN allowed multiple causes to be assigned to each case. Although we examined a relatively large study population, sample size was limited for some comparisons, especially the most obese women. To improve statistical efficiency, we examined BMI specified as a continuous variable, in addition to examining traditional BMI categories. Given this sparsity of data, however, results should be extrapolated to the highest levels of BMI with caution. Another limitation is the exclusion of potentially relevant covariates. For example, maternal weight gain during pregnancy and post-partum weight retention (the latter relevant only to multiparous women) could confound or modify the association between maternal pre-pregnancy BMI and risk of stillbirth. Their investigation is important but beyond the scope of the current analyses; their consideration is analytically complex due to their correlation with gestational duration and inter-pregnancy interval [21]. Smoking is also an important risk factor for stillbirth; we did not include it because cigarette smoke exposure is not well-captured by the birth certificate [22].

In summary, increasing obesity was associated with increasing risk of stillbirth. Risks for a 10-unit increase in BMI, which corresponds approximately to the difference in BMI between a normal-weight and moderately obese woman, tended to increase 1.5- to 2.0-fold. The strength of association tended to be even stronger with larger differences in BMI and to vary based on factors such as gestational age, race-ethnicity or parity, or underlying cause of death. Given the nature of the available data, we were unable to determine contributing mechanisms, but they are likely diverse. It is critical to understand these mechanisms in order to more fully understand the etiologies of stillbirth and how to prevent it. These findings are important for identifying women at risk of having a stillbirth in the clinical setting, especially given the high and increasing population prevalence of obesity and the strength of the observed associations.

## Supporting Information

S1 Table Association of stillbirth with maternal pre-pregnancy body mass index specified as a continuous variable, by gestational age, race-ethnicity and parity, California 2007–2010, and including women with diabetes or hypertensive disorders. (DOC)

S2 Table Association of stillbirth with maternal pre-pregnancy body mass index specified as a continuous variable, by gestational age, race-ethnicity and parity, California 2007–2010, among stillbirths that were due to (a) obstetric or (b) unknown causes. (DOC)
