# Supplementary material for: Prepregnancy Obesity and Risks of Stillbirth
Source: PLoS One. 2015 Oct 14;10(10):e0138549. doi: 10.1371/journal.pone.0138549 (PMC4605840; doi:10.1371/journal.pone.0138549)
Supplement: S1 Table — (DOC) [file pone.0138549.s001.doc]

**Supplementary Table 1.** Association of stillbirth with maternal pre-pregnancy body mass index specified as a continuous variable, by gestational age, race-ethnicity and parity, California 2007-2010, and including women with diabetes or hypertensive disorders.

| Race/ethnicity | Change in BMI | 20 -23 weeks | 24-27 weeks | 28-31 weeks | 32-36 weeks | 37-41 weeks |
| --- | --- | --- | --- | --- | --- | --- |
|  |  | RR (CI) | RR (CI) | RR (CI) | RR (CI) | RR (CI) |
| **NULLIPAROUS** |  |  |  |  |  |  |
| Non-Hispanic White | 1-unit RR | **1.05 (1.03,1.07)** | 1.03 (1.00,1.06) | **1.04 (1.01,1.07)** | 1.02 (0.99,1.05) | **1.06 (1.04,1.08)** |
|  | 5-unit | **1.27 (1.13,1.42)** | 1.16 (0.99,1.36) | **1.22 (1.04,1.43)** | 1.11 (0.96,1.29) | **1.33 (1.20,1.47)** |
|  | 10-unit | **1.62 (1.29,2.03)** | 1.35 (0.97,1.86) | **1.48 (1.08,2.05)** | 1.24 (0.93,1.66) | **1.76 (1.43,2.17)** |
|  | 15-unit | **2.05 (1.46,2.89)** | 1.56 (0.96,2.54) | **1.81 (1.12,2.93)** | 1.38 (0.89,2.14) | **2.34 (1.72,3.20)** |
|  | 20-unit | **2.61 (1.66,4.11)** | 1.81 (0.95,3.46) | **2.20 (1.16,4.20)** | 1.54 (0.86,2.75) | **3.11 (2.06,4.71)** |
| Non-Hispanic Black | 1-unit RR | 1.03 (1.00,1.06) | 1.02 (0.99,1.06) | **1.05 (1.01,1.08)** | **1.05 (1.02,1.08)** | **1.05 (1.01,1.08)** |
|  | 5-unit | 1.15 (1.00,1.32) | 1.12 (0.93,1.34) | **1.25 (1.04,1.50)** | **1.29 (1.12,1.50)** | **1.26 (1.07,1.49)** |
|  | 10-unit | 1.33 (1.00,1.75) | 1.25 (0.87,1.80) | **1.57 (1.09,2.26)** | **1.67 (1.24,2.25)** | **1.59 (1.14,2.23)** |
|  | 15-unit | 1.53 (1.00,2.32) | 1.40 (0.81,2.41) | **1.96 (1.14,3.40)** | **2.16 (1.39,3.38)** | **2.01 (1.21,3.34)** |
|  | 20-unit | 1.76 (1.00,3.08) | 1.56 (0.75,3.23) | **2.46 (1.19,5.10)** | **2.80 (1.55,5.06)** | **2.54 (1.29,5.00)** |
| Hispanic | 1-unit RR | **1.08 (1.06,1.09)** | 1.01 (0.98,1.04) | 1.03 (1.00,1.06) | **1.03 (1.00,1.05)** | **1.04 (1.02,1.07)** |
|  | 5-unit | **1.44 (1.34,1.55)** | 1.07 (0.92,1.24) | 1.14 (0.99,1.32) | **1.15 (1.02,1.29)** | **1.24 (1.12,1.38)** |
|  | 10-unit | **2.08 (1.79,2.41)** | 1.14 (0.85,1.54) | 1.31 (0.99,1.73) | **1.32 (1.05,1.65)** | **1.54 (1.26,1.89)** |
|  | 15-unit | **3.00 (2.40,3.73)** | 1.22 (0.78,1.90) | 1.49 (0.98,2.27) | **1.51 (1.07,2.12)** | **1.92 (1.41,2.60)** |
|  | 20-unit | **4.32 (3.22,5.79)** | 1.30 (0.72,2.36) | 1.71 (0.97,2.99) | **1.73 (1.10,2.73)** | **2.38 (1.58,3.58)** |
| **MULTIPAROUS** |  |  |  |  |  |  |
| Non-Hispanic White | 1-unit RR | **1.03 (1.01,1.05)** | **1.05 (1.03,1.08)** | **1.12 (1.05,1.19)^2^** | **1.03 (1.01,1.05)** | **1.05 (1.03,1.07)** |
|  | 5-unit | **1.15 (1.03,1.30)** | **1.29 (1.14,1.46)** | **1.63 (1.25,2.13)** | **1.16 (1.03,1.30)** | **1.28 (1.16,1.42)** |
|  | 10-unit | **1.33 (1.05,1.68)** | **1.66 (1.29,2.13)** | **2.26 (1.51,3.38)** | **1.34 (1.06,1.69)** | **1.64 (1.33,2.01)** |
|  | 15-unit | **1.54 (1.08,2.18)** | **2.13 (1.47,3.10)** | **2.64 (1.63,4.25)** | **1.55 (1.09,2.20)** | **2.10 (1.54,2.85)** |
|  | 20-unit | **1.77 (1.11,2.83)** | **2.75 (1.67,4.52)** | **2.61 (1.38,4.94)** | **1.79 (1.13,2.86)** | **2.68 (1.78,4.04)** |
| Non-Hispanic Black | 1-unit RR | **1.11 (1.03,1.19)^2^** | 1.03 (0.99,1.06) | 1.02 (0.98,1.05) | **1.04 (1.01,1.07)** | **1.14 (1.03,1.26)^2^** |
|  | 5-unit | **1.57 (1.16,2.11)** | 1.15 (0.97,1.35) | 1.08 (0.91,1.29) | **1.22 (1.08,1.38)** | **1.69 (1.14,2.49)** |
|  | 10-unit | **2.11 (1.32,3.37)** | 1.32 (0.94,1.84) | 1.16 (0.82,1.66) | **1.48 (1.16,1.90)** | **2.03 (1.15,3.58)** |
|  | 15-unit | **2.45 (1.42,4.21)** | 1.51 (0.92,2.49) | 1.26 (0.74,2.13) | **1.81 (1.25,2.62)** | **1.75 (0.90,3.39)** |
|  | 20-unit | **2.44 (1.33,4.46)** | 1.73 (0.89,3.37) | 1.36 (0.67,2.74) | **2.20 (1.34,3.61)** | **1.08 (0.42,2.78)** |
| Hispanic | 1-unit RR | **1.03 (1.01,1.04)** | 1.00 (0.98,1.02) | 1.02 (1.00,1.04) | **0.99 (0.96,1.02)^2^** | **1.04 (1.02,1.05)** |
|  | 5-unit | **1.16 (1.07,1.24)** | 1.01 (0.91,1.13) | 1.10 (0.99,1.22) | **0.97 (0.86,1.09)** | **1.20 (1.12,1.29)** |
|  | 10-unit | **1.33 (1.15,1.55)** | 1.02 (0.82,1.28) | 1.20 (0.98,1.48) | **0.99 (0.82,1.20)** | **1.44 (1.25,1.65)** |
|  | 15-unit | **1.54 (1.23,1.93)** | 1.03 (0.74,1.44) | 1.32 (0.97,1.79) | **1.08 (0.85,1.37)** | **1.73 (1.40,2.13)** |
|  | 20-unit | **1.78 (1.32,2.40)** | 1.05 (0.67,1.63) | 1.44 (0.95,2.18) | **1.25 (0.93,1.67)** | **2.08 (1.57,2.74)** |

^1^ Relative Risks (RR) reflect estimated risk of stillbirth relative to term (37-41 weeks) live birth adjusted for maternal age, education, and height. Each 5-unit change in BMI reflects the approximate difference in risk between the following categories of BMI: a 5-unit change represents the approximate difference in risk between women with normal BMI (18.5-24.9 kg/m^2^, with 22.5 taken as the approximate mid-point) versus overweight (25.0-29.9 kg/m^2^, with 27.5 as the mid-point); a 10-unit change, the difference between women with normal BMI and obese class I (BMI 30.0-34.9 kg/m^2^, with 32.5 as the mid-point); a 15-unit change, the difference between normal BMI and obese class II (BMI 35.0-39.9 kg/m^2^, with 37.5 as mid-point); and a 20-unit change, the difference between normal BMI and obese class III (BMI ≥40.0 kg/m^2^, with 42.5 as reference).

^2^ The quadratic term (BMI-squared) was significant (p<0.10) for the noted models; this is an indication that the association between BMI and risk of stillbirth was not linear, as reflected in the RRs for these groups, which are all relative to a BMI of 22.5 kg/m^2^.
