# Supplementary material for: Prepregnancy Obesity and Risks of Stillbirth
Source: PLoS One. 2015 Oct 14;10(10):e0138549. doi: 10.1371/journal.pone.0138549 (PMC4605840; doi:10.1371/journal.pone.0138549)
Supplement: S2 Table — (DOC) [file pone.0138549.s002.doc]

**Supplementary Table 2.** Association of stillbirth with maternal pre-pregnancy body mass index specified as a continuous variable, by gestational age, race-ethnicity and parity, California 2007-2010, among stillbirths that were due to (a) obstetric or (b) unknown causes.^1^

(a)

| Race/ethnicity | Change in BMI | 20 -23 weeks | 24-27 weeks | 28-31 weeks | 32-36 weeks | 37-41 weeks |
| --- | --- | --- | --- | --- | --- | --- |
|  |  | RR (CI) | RR (CI) | RR (CI) | RR (CI) | RR (CI) |
| **NULLIPAROUS**  Non-Hispanic White | 1-unit | 1.09 (1.06,1.13) | 1.07 (0.98,1.17) | NC | NC | NC |
|  | 5-unit | 1.55 (1.33,1.82) | 1.40 (0.88,2.22) | NC | NC | NC |
|  | 10-unit | 2.42 (1.76,3.31) | 1.96 (0.78,4.93) | NC | NC | NC |
|  | 15-unit | 3.76 (2.34,6.03) | 2.75 (0.69,11.0) | NC | NC | NC |
|  | 20-unit | 5.84 (3.11,11.0) | 3.86 (0.61,24.3) | NC | NC | NC |
| Non-Hispanic Black | 1-unit | 1.08 (1.04,1.11) | NC | NC | NC | NC |
|  | 5-unit | 1.44 (1.22,1.69) | NC | NC | NC | NC |
|  | 10-unit | 2.07 (1.50,2.86) | NC | NC | NC | NC |
|  | 15-unit | 2.98 (1.83,4.84) | NC | NC | NC | NC |
|  | 20-unit | 4.29 (2.25,8.18) | NC | NC | NC | NC |
| Hispanic | 1-unit | 1.10 (1.08,1.12) | 1.05 (0.98,1.14) | 1.01 (0.91,1.11) | NC | 1.06 (0.97,1.16) |
|  | 5-unit | 1.60 (1.46,1.76) | 1.31 (0.90,1.89) | 1.04 (0.64,1.70) | NC | 1.35 (0.85,2.12) |
|  | 10-unit | 2.57 (2.13,3.09) | 1.70 (0.81,3.57) | 1.08 (0.40,2.87) | NC | 1.82 (0.73,4.51) |
|  | 15-unit | 4.11 (3.11,5.44) | 2.23 (0.73,6.75) | 1.12 (0.26,4.87) | NC | 2.45 (0.62,9.58) |
|  | 20-unit | 6.59 (4.54,9.57) | 2.91 (0.66,12.8) | 1.16 (0.16,8.26) | NC | 3.29 (0.53,20.3) |
|  |  |  |  |  |  |  |
| **MULTIPAROUS**  Non-Hispanic White | 1-unit | 1.07 (1.04,1.11) | 1.09 (1.03,1.16) | NC | NC | NC |
|  | 5-unit | 1.42 (1.19,1.69) | 1.55 (1.14,2.11) | NC | NC | NC |
|  | 10-unit | 2.02 (1.42,2.87) | 2.40 (1.30,4.43) | NC | NC | NC |
|  | 15-unit | 2.87 (1.70,4.87) | 3.71 (1.47,9.33) | NC | NC | NC |
|  | 20-unit | 4.09 (2.02,8.25) | 5.74 (1.68,19.6) | NC | NC | NC |
| Non-Hispanic Black | 1-unit | 1.04 (1.00,1.09) | 1.01 (0.92,1.11) | NC | NC | NC |
|  | 5-unit | 1.24 (1.00,1.52) | 1.05 (0.66,1.67) | NC | NC | NC |
|  | 10-unit | 1.53 (1.01,2.32) | 1.11 (0.44,2.79) | NC | NC | NC |
|  | 15-unit | 1.89 (1.01,3.53) | 1.17 (0.29,4.65) | NC | NC | NC |
|  | 20-unit | 2.33 (1.01,5.38) | 1.23 (0.19,7.76) | NC | NC | NC |
| Hispanic | 1-unit | 1.04 (1.01,1.06) | 0.97 (0.90,1.05) | 1.04 (0.96,1.13) | 1.00 (0.91,1.09) | 1.05 (0.97,1.14) |
|  | 5-unit | 1.20 (1.07,1.33) | 0.86 (0.59,1.25) | 1.22 (0.80,1.86) | 0.98 (0.62,1.54) | 1.28 (0.85,1.94) |
|  | 10-unit | 1.43 (1.15,1.78) | 0.74 (0.35,1.56) | 1.49 (0.64,3.45) | 0.96 (0.39,2.38) | 1.64 (0.72,3.75) |
|  | 15-unit | 1.71 (1.24,2.37) | 0.64 (0.21,1.95) | 1.82 (0.51,6.42) | 0.95 (0.24,3.67) | 2.11 (0.61,7.25) |
|  | 20-unit | 2.05 (1.33,3.16) | 0.55 (0.12,2.43) | 2.21 (0.41,11.9) | 0.93 (0.15,5.67) | 2.70 (0.52,14.0) |

(b)

| Race/ethnicity | Change in BMI | 20 -23 weeks | 24-27 weeks | 28-31 weeks | 32-36 weeks | 37-41 weeks |
| --- | --- | --- | --- | --- | --- | --- |
|  |  | RR (CI) | RR (CI) | RR (CI) | RR (CI) | RR (CI) |
| **NULLIPAROUS**  Non-Hispanic White | 1-unit | 1.05 (0.99,1.10) | 1.00 (0.92,1.07) | 1.03 (0.97,1.10) | 1.02 (0.97,1.08) | 1.05 (1.01,1.10) |
|  | 5-unit | 1.26 (0.96,1.64) | 0.98 (0.67,1.42) | 1.18 (0.85,1.62) | 1.11 (0.84,1.46) | 1.30 (1.05,1.62) |
|  | 10-unit | 1.58 (0.93,2.68) | 0.95 (0.45,2.02) | 1.38 (0.73,2.62) | 1.22 (0.71,2.12) | 1.69 (1.10,2.61) |
|  | 15-unit | 1.98 (0.90,4.40) | 0.93 (0.30,2.88) | 1.63 (0.62,4.25) | 1.35 (0.59,3.09) | 2.21 (1.15,4.22) |
|  | 20-unit | 2.49 (0.86,7.20) | 0.91 (0.20,4.10) | 1.92 (0.53,6.89) | 1.50 (0.50,4.50) | 2.87 (1.21,6.81) |
| Non-Hispanic Black | 1-unit | NC | 1.02 (0.95,1.10) | 1.08 (1.01,1.15) | 1.05 (0.99,1.12) | 1.04 (0.97,1.11) |
|  | 5-unit | NC | 1.11 (0.76,1.62) | 1.46 (1.06,2.01) | 1.28 (0.94,1.74) | 1.19 (0.86,1.65) |
|  | 10-unit | NC | 1.24 (0.58,2.62) | 2.13 (1.12,4.02) | 1.63 (0.89,3.01) | 1.42 (0.74,2.72) |
|  | 15-unit | NC | 1.38 (0.45,4.25) | 3.10 (1.19,8.06) | 2.09 (0.83,5.23) | 1.70 (0.64,4.48) |
|  | 20-unit | NC | 1.53 (0.34,6.88) | 4.52 (1.27,16.2) | 2.67 (0.78,9.07) | 2.02 (0.55,7.38) |
| Hispanic | 1-unit | 1.07 (1.02,1.11) | 1.01 (0.95,1.06) | 1.00 (0.94,1.06) | 1.01 (0.96,1.05) | 1.03 (0.99,1.07) |
|  | 5-unit | 1.37 (1.11,1.69) | 1.03 (0.79,1.36) | 1.00 (0.74,1.34) | 1.03 (0.83,1.29) | 1.16 (0.95,1.42) |
|  | 10-unit | 1.88 (1.24,2.86) | 1.07 (0.62,1.86) | 1.00 (0.55,1.80) | 1.07 (0.69,1.66) | 1.34 (0.89,2.01) |
|  | 15-unit | 2.59 (1.39,4.83) | 1.11 (0.48,2.53) | 1.00 (0.41,2.41) | 1.10 (0.57,2.14) | 1.55 (0.85,2.85) |
|  | 20-unit | 3.55 (1.54,8.17) | 1.14 (0.38,3.44) | 1.00 (0.31,3.23) | 1.14 (0.47,2.76) | 1.80 (0.80,4.04) |
|  |  |  |  |  |  |  |
| **MULTIPAROUS**  Non-Hispanic White | 1-unit | 0.98 (0.91,1.04) | 1.03 (0.98,1.08) | 1.09 (1.04,1.14) | 1.06 (1.02,1.11) | 1.06 (1.02,1.10) |
|  | 5-unit | 0.88 (0.63,1.24) | 1.15 (0.89,1.48) | 1.51 (1.20,1.91) | 1.37 (1.11,1.69) | 1.34 (1.11,1.62) |
|  | 10-unit | 0.78 (0.40,1.53) | 1.32 (0.80,2.19) | 2.29 (1.43,3.65) | 1.87 (1.22,2.85) | 1.80 (1.23,2.63) |
|  | 15-unit | 0.69 (0.25,1.90) | 1.51 (0.71,3.23) | 3.46 (1.71,6.98) | 2.55 (1.35,4.81) | 2.41 (1.36,4.26) |
|  | 20-unit | 0.61 (0.16,2.35) | 1.74 (0.63,4.78) | 5.23 (2.05,13.4) | 3.48 (1.50,8.11) | 3.23 (1.51,6.90) |
| Non-Hispanic Black | 1-unit | 1.02 (0.95,1.09) | 1.01 (0.92,1.10) | 1.04 (0.98,1.11) | 1.01 (0.95,1.07) | 1.01 (0.93,1.09) |
|  | 5-unit | 1.10 (0.77,1.57) | 1.04 (0.68,1.59) | 1.23 (0.89,1.71) | 1.06 (0.79,1.42) | 1.03 (0.69,1.52) |
|  | 10-unit | 1.21 (0.59,2.47) | 1.07 (0.46,2.52) | 1.52 (0.79,2.93) | 1.12 (0.63,2.01) | 1.06 (0.48,2.33) |
|  | 15-unit | 1.32 (0.45,3.89) | 1.11 (0.31,3.99) | 1.88 (0.71,5.00) | 1.19 (0.50,2.84) | 1.09 (0.33,3.55) |
|  | 20-unit | 1.45 (0.35,6.11) | 1.15 (0.21,6.33) | 2.32 (0.63,8.56) | 1.26 (0.40,4.03) | 1.12 (0.23,5.41) |
| Hispanic | 1-unit | 1.00 (0.96,1.05) | 1.01 (0.97,1.05) | 1.00 (0.96,1.05) | 1.00 (0.97,1.04) | 1.03 (1.00,1.06) |
|  | 5-unit | 1.02 (0.84,1.25) | 1.03 (0.84,1.26) | 1.01 (0.81,1.26) | 1.02 (0.87,1.20) | 1.17 (1.02,1.34) |
|  | 10-unit | 1.05 (0.70,1.57) | 1.07 (0.71,1.60) | 1.02 (0.66,1.58) | 1.05 (0.76,1.45) | 1.36 (1.03,1.79) |
|  | 15-unit | 1.07 (0.58,1.97) | 1.10 (0.60,2.01) | 1.03 (0.53,1.98) | 1.07 (0.66,1.74) | 1.59 (1.05,2.40) |
|  | 20-unit | 1.10 (0.49,2.48) | 1.14 (0.51,2.54) | 1.04 (0.43,2.49) | 1.10 (0.58,2.09) | 1.85 (1.06,3.21) |

^1^ Relative Risks (RR) reflect estimated risk of stillbirth relative to term (37-41 weeks) live birth adjusted for maternal age, education, and height. Each 5-unit change in BMI reflects the approximate difference in risk between the following categories of BMI: a 5-unit change represents the approximate difference in risk between women with normal BMI (18.5-24.9 kg/m^2^, with 22.5 taken as the approximate mid-point) versus overweight (25.0-29.9 kg/m^2^, with 27.5 as the mid-point); a 10-unit change, the difference between women with normal BMI and obese class I (BMI 30.0-34.9 kg/m^2^, with 32.5 as the mid-point); a 15-unit change, the difference between normal BMI and obese class II (BMI 35.0-39.9 kg/m^2^, with 27.5 as mid-point); and a 20-unit change, the difference between normal BMI and obese class III (BMI ≥40.0 kg/m^2^, with 42.5 as reference). Analyses exclude women with gestational or pre-gestational diabetes or pregnancy-induced or chronic hypertension with the exception of models for stillbirths at 20-23 weeks.

**NC** = not calculated; RR’s are not shown for comparisons that involved fewer than 10 cases.
